# An American View of English Ways

**Published:** 1906-12-01

**Authors:** J. Horner


### EDITOR'S LETTER-BOX.

[Our Correspondents are reminded that prolixity is a great bar to publication, and that brevity of style and conciseness of statement greatly facilitate early insertion.]

### AN AMERICAN VIEW OF ENGLISH WAYS.

Sir,—Of course it is to be expected that a doctor's paid organ should stick up for the doctors. I mean your advocating larger salaries for ships' doctors. You think £100 a year and perquisites not enough. England is nothing but a nation of "graft" from King Edward VII. down. Look at your endowed hospitals giving doctors large salaries. Look at police surgeons with large salaries to treat healthy policemen. Look at the Prince of Wales drawing an admiral's salary and vast money besides, and doing absolutely nothing for it. Some of these ships' doctors kill more than they cure. Look at the Royal yacht being used for the King of Norway.

31 Mulberry Street, Detroit, U.S.A. J. Horner.

*** Our correspondent ought to have concluded: "Look at the ignorance I have displayed on the matters I have mentioned." Fiction could take no wilder flight than our correspondent has permitted his imagination through the region of English facts.—Ed. T. H.

An "Anonymous" donor has sent £100 to the Cancer Research Fund of the Middlesex Hospital.

